# 5-Azacytidine: A Promoter of Epigenetic Changes in the Quest to Improve Plant Somatic Embryogenesis

**DOI:** 10.3390/ijms19103182

**Published:** 2018-10-16

**Authors:** Pedro Osorio-Montalvo, Luis Sáenz-Carbonell, Clelia De-la-Peña

**Affiliations:** Unidad de Biotecnología, Centro de Investigación Científica de Yucatán, Calle 43 No. 130 x 32 y 34, Col. Chuburná de Hidalgo, 97205 Mérida, Yucatán, Mexico; jacket_147@hotmail.com (P.O.-M.); vyca@cicy.mx (L.S.-C.)

**Keywords:** somatic embryogenesis, DNA methylation, 5-Azacytidine, epigenetics, hypomethylation, plant tissue culture, 2,4-dichlorophenoxyacetic acid (2,4-D), in vitro

## Abstract

Somatic embryogenesis (SE) is a widely studied process due to its biotechnological potential to generate large quantities of plants in short time frames and from different sources of explants. The success of SE depends on many factors, such as the nature of the explant, the microenvironment generated by in vitro culture conditions, and the regulation of gene expression, among others. Epigenetics has recently been identified as an important factor influencing SE outcome. DNA methylation is one of the most studied epigenetic mechanisms due to its essential role in gene expression, and its participation in SE is crucial. DNA methylation levels can be modified through the use of drugs such as 5-Azacytidine (5-AzaC), an inhibitor of DNA methylation, which has been used during SE protocols. The balance between hypomethylation and hypermethylation seems to be the key to SE success. Here, we discuss the most prominent recent research on the role of 5-AzaC in the regulation of DNA methylation, highlighting its importance during the SE process. Also, the molecular implications that this inhibitor might have for the increase or decrease in the embryogenic potential of various explants are reviewed.

## 1. Introduction

With more than seven billion people to feed, the need for food and energy has become an urgent problem to resolve. The worldwide population needs healthier and safer food, and one way to do this is by increasing crop production. Plant tissue culture (PTC) is a set of biotechnological techniques to establish, maintain and multiply cells, tissues, organs and even whole plants under controlled and aseptic conditions to generate more plants in a shorter period than conventional methods. PTC has been used with great success in the agricultural industry to produce fruit, ornamental and medicinal plants, and even forest-dwelling species. Therefore, PTC represents the most promising area of application today and offers a perspective for the future [1].

Two of the most common PTC methods for the regeneration of plant structures are organogenesis and somatic embryogenesis (SE). Organogenesis refers to the generation of monopolar structures (shoots, leaves or roots) that may arise directly from the meristem or indirectly from the undifferentiated cell masses called callus [2]. On the other hand, SE is a process that generates bipolar structures (shoot apical meristem and root apical meristem), similar to a zygotic embryo, developed from a non-zygotic cell with no vascular connection to the original tissue [3,4]. Both organogenesis and SE can be obtained directly or indirectly; it means that organs or somatic embryos can be developed directly from the explant or the callus, respectively [5]. Regardless of the morphogenic pathway (direct or indirect), SE can produce genetically identical individuals to the donor of the explant, which is a significant advantage in when controlling the quality and selection of plants [6]. It is important to highlight that for study purposes, and from an economic point of view, it is necessary to have the capacity to differentiate between different callus types, mainly embryogenic callus (callus that generates somatic embryos [7]) and non-embryogenic callus (callus that does not generate somatic embryos). By being able to identify the embryogenic callus from the non-embryogenic ones, we avoid selecting callus under in vitro conditions that will not generate the necessary number of somatic embryos.

Thanks to the study of SE, it has been possible to isolate genes, proteins, and metabolites involved in the process of cell differentiation. These discoveries have led to a better understanding of differentiation, as well as the genetic mechanisms involved in the transition from one stage to the next. It has highlighted the importance of continuing to study SE in all aspects (biochemical, genetic and transcriptomic) to accelerate the discovery, isolation, and characterization of genes involved in different cellular processes [8,9,10].

**Figure 1:**
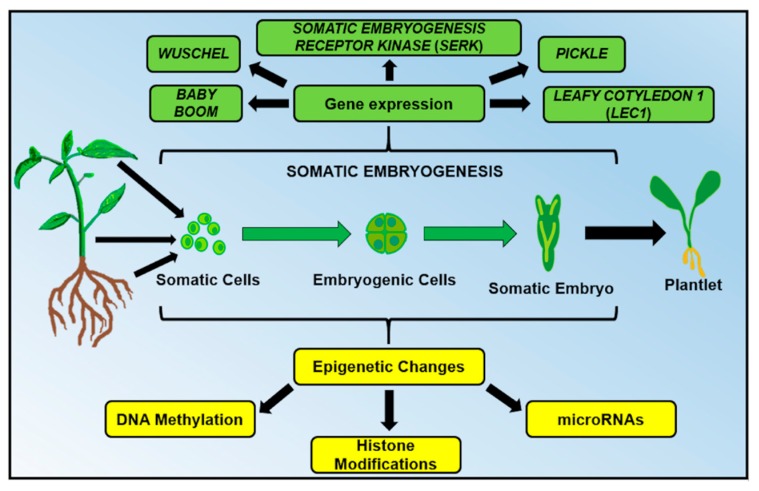
In plant somatic embryogenesis, genetic (green) and epigenetic (yellow) mechanisms induce the development of embryogenic cells from any explant (cells isolated from the leaves, shoots or roots). Due to the totipotentiality properties of plant cells, a group of embryogenic cells can develop into a complete and functional plantlet.

SE can be triggered in the explant by applying specific conditions such as stress (mechanical, osmotic, chemical, heavy metals, hypoxia, temperature and ultraviolet light) as well as by both endogenous and exogenous plant growth regulator (PGR) levels [11,12,13]. The PGR most used to induce SE is the exogenous auxin 2,4-Dichlorophenoxyacetic acid (2,4-D) [14]. The different conditions (endogenous or exogenous to the explant) can influence the metabolic patterns, gene expression and epigenetic mechanisms (DNA methylation, histone modifications, and microRNAs) of the explant, provoking a cell (or a group of cells) to change its nature (Figure 1). A somatic cell can develop into a totipotent, undifferentiated and embryogenic cell and the embryogenic cell can generate all the cells forming a somatic embryo, which later becomes a complete and functional plantlet (Figure 1).

**Figure 2:**
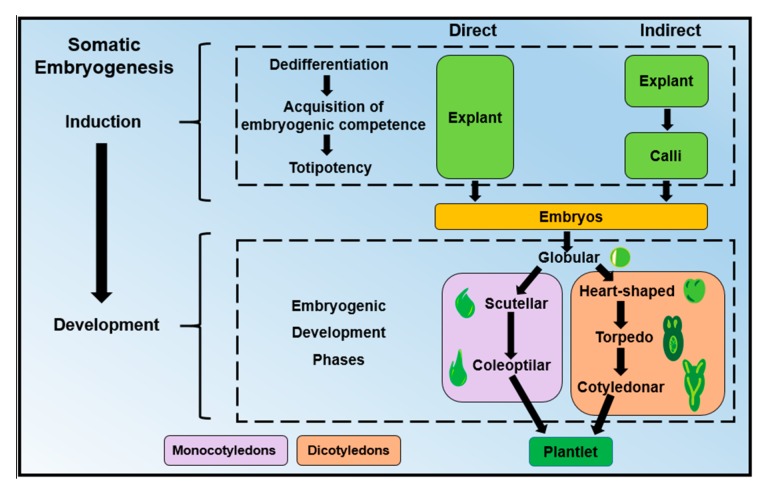
Differences between direct somatic embryogenesis (SE) and indirect SE during the induction and developmental stages in dicotyledonous and monocotyledonous plants.

The SE process can be divided into two significant steps: induction and development (Figure 2). The induction stage has three phases: dedifferentiation, totipotency, and acquisition of the embryogenic competence [15]. In the dedifferentiation step, mature explant cells lose their specific fate to become meristematic cells. In the totipotency step, the cells acquire the potential to generate any plant cell [16]. In the last step, the acquisition phase of embryogenic competence, the cells reach a state of somatic-embryogenic transition and only require a minimum exogenously applied stimulus to become an embryogenic cell [17]. Morphologically, competent cells already show similar characteristics to those in meristematic cells or zygotes, such as small size and rounded shape with abundant cytoplasm and small vacuoles [18]. Next follows the second major stage of the SE process, in which the establishment of the first proembriogenic phases initiates with the transition of the embryogenic forms. These forms are different in dicotyledons and monocotyledons. For instance, in dicotyledonous plants, the embryogenic structures are globular, heart-shaped, torpedo and cotyledonar, while those of monocotyledonous plants are globular, scutellar and coleoptilar (Figure 2).

In recent years, numerous reports have demonstrated that SE is a process strongly regulated by epigenetic mechanisms (DNA methylation, posttranslational modifications of histones and miRNAs), which induce chromatin remodeling [7,11,19]. DNA methylation and histone modification are epigenetic changes that can reorganize chromatin architecture during the in vitro culture of plants [20,21]. Since the 1980’s, it has been proposed and reported that embryogenic capacity could be conditioned by DNA methylation levels [22]. Currently, it is known that DNA methylation plays an essential role in the regulation of gene expression during development. Methylation states in the DNA, resulting from in vitro cultures, are often related to the control of SE and the regeneration process through the modulation of gene expression [19,23]. By changing the methylation profile, it is possible to alter gene expression, and this can be applied to produce a large number of high-quality plants or to improve the agronomic characteristics leading to the improvement of a crop [24]. By understanding how methylation alterations influence the acquisition of the developmental cell fate during in vitro cultures, we would be able to develop new strategies to enhance the embryogenic capability and totipotency in recalcitrant plant species and genotypes.

As early as the 1980s, an essential role in the control of gene expression was attributed to 5-methylcytosine [25]; thereafter, the focus was on molecular analogs to methyl derivatives of cytosine such as 5-Azacytidine (5-AzaC). 5-AzaC was first used as an anticancer agent, acting as an analog of cytidine and incorporated into DNA, where, due to the nitrogen at the 5′-position of the pyrimidine ring, it could not be methylated [26,27]. The first pieces of evidence that 5-AzaC could induce the expression of silenced genes was carried out on transformed avian and animal virus cell lines. It was observed that when the animal cells (different tissues of mouse and chicken) were treated with this drug, DNA demethylation was detected a short time later (48–96 h) [28,29,30]. At that time, it was suggested that 5-AzaC was incorporated into DNA, causing methylation to be inhibited and positively affecting gene expression and differentiation [26]. However, there is evidence that this analog might be incorporated into DNA to cause demethylation and, in the first round of replication, lead to the appearance of hemimethylated DNA. Only after the second round of DNA replication do completely unmethylated strands appear through the action of the methylation maintenance system. In fact, a 5-AzaC residue incorporated into DNA leads to vast stretches of DNA becoming unmethylated, apparently due to long-term inhibition of the 5-DNA-methyltransferase enzymes and its mode of processing action [26,31,32]. There are other DNA hypomethylating analogs such as ethionine, 2-amino-5-ethoxycarbonyl-pyrimidine-4 (3H) (ECP) [21], and zebularine [33]. Currently, the most widely used and studied hypomethylating drug is 5-AzaC, and very interesting studies are being carried out to study its effects and the relationship it has on the expression of genes that are important for the SE process [34]. In this review, we will discuss the most recent and important works that have used 5-AzaC to modify the methylation patterns in SE protocols to increase the embryogenic potential and at the same time understand the effect of methylation levels on this process.

## 2. DNA Methylation: A Key Player During Somatic Embryogenesis

It is widely known that embryogenic potential is higher in explants obtained from young tissue constituted mostly of cells with a low level of differentiation, such as zygotic embryos or meristems, unlike other tissues composed of well-differentiated cells such as fundamental, conduction or epidermic tissues [4,35,36,37]. A couple of years ago, it was revealed that the state of differentiation of plant tissues is strongly controlled by DNA methylation [38], so that the embryogenic or non-embryogenic response between both types of tissues could be determined epigenetically.

DNA methylation is an epigenetic mechanism that plays critical roles in genome integrity, genomic imprinting, X chromosome inactivation, suppression of transposons and retroviruses, and gene expression [39,40,41,42,43]. DNA methylation occurs after DNA synthesis, and it is catalyzed by enzymes known as DNA cytosine methyltransferases (DCMTases). DCMTases transfer a methyl group of the S-adenosyl-L-methionine molecule to the carbon 5′ of the pyrimidine ring of the cytosines. In mammals, this methylation occurs only in cytosines adjacent to guanines (CpG) [44]. However, in plants, the methylation not only occurs at the CpG sites but also in the symmetric CpHpG and asymmetric CpHpH sequences (where H is A, T or C) [45]. Based on sequence homology within the enzyme′s C-terminal catalytic domains, most DCMTases can be grouped into four distinct families, omitting fungal DCMTases [46]. Plants have four classes of DCMTases: Methyltransferase (MET), Domains Rearranged Methyltransferase (DRM), DNA Nucleotide Methyltransferase 2 (DNMT2) and Chromomethylase (CMT, appearing to be unique to plants), while other eukaryotic organisms have only two or three classes [21]. Additionally, there are two types of methylation: maintenance: (1) this occurs in hemimethylated sites, and is inherited from generation to generation and (2) *de novo*, which arises spontaneously in places where there was no methylation before [47].

MET1 (DNMT1, ortholog in animals) catalyzes both maintenance and de novo methylation at CpG sites, while that CMT3 catalyzes maintenance methylation at CpHpG and CpHpH sites [48,49,50,51]. In addition, DRM2 (DNMT3A, ortholog in animals) catalyzes de novo methylation at CpG, CpHpG and CpHpH sites, and is related to the RdDM mechanism—RNA-directed DNA methylation [41,49]. It has been reported that although Arabidopsis *met1* mutant plants are viable, they are entirely lacking in CpG methylation [41,45,52,53,54]. DRM2 and DNMT3A structures are not identical to each other, and yet the overall folding is similar in both enzymes, which could explain the structure’s conservation in its functions in both plants and animals [43]. The fact that the plant-only DRM2 contains a rearrangement similar to a DNMT3A in its catalytic domain suggests that this rearrangement may have occurred during the early stages of plant evolution [55]. In *Arabidopsis thaliana*, the impact of DNA methylation on SE was analyzed [34], and it was found that a decrease in global DNA methylation (GDM) during SE contrasted with the positive regulation of the genes *MET1* and *CMT3* that codify DNA methylases, and the down-regulation of genes *ROS1*, *DME* and *DML2* (DNA demethylases). Therefore, the level of GDM seemed to correlate with the transcriptional activity of the coding genes of DNA methylases/demethylases.

### 2.1. Hypomethylation Promotes Embryogenic Capacity

DNA methylation plays a vital role in cell dedifferentiation, redifferentiation and the growth and development of plants [56]. In SE, DNA methylation regulates and maintains the gene expression programs of several genes; however, due to the previously reported critical implications for the embryogenic process in plants, a group of genes has been especially studied (Figure 1): *Leafy Cotyledon 1* (*LEC1*), *Wuschel* (*WUS), Somatic Embryogenesis Receptor Kinase* (*SERK*), *Pickle* (*PKL*) and *Baby Boom* (*BBM*) [20,23,57]. For instance, the manipulation of methylation in specific genes, such as *LEC1* [58] and *Wuschel* (*WUS*) [59], drastically affects the regulation of the differentiation of plant explants. In the SE process of *Coffea canephora*, it was reported that between 21 and 28 days after induction (dai) there was a reduction of GDM that coincided with the formation of the first proembryogenic masses. Subsequently, a gradual increase of GDM was observed between 28 and 49 dai. During this same period, somatic embryos appeared and developed from the globular stage to the torpedo stage. Finally, the pronounced increase in GDM between 49 and 56 dai coincided with the transition from somatic embryos in the torpedo phase to the cotyledon phase. These observations support the idea that a decrease in global DNA methylation could be a critical step in triggering cell dedifferentiation and acquiring cell totipotency in somatic cells. We can also suggest that the epigenetic cell reprogramming through dynamic changes in DNA methylation promotes the embryogenic route and the development of somatic embryos [60].

In *Boesenbergia rotunda* (L.) Mansf., the expression of the *SERK*, *BBM*, *LEC2* and *WUS* genes was studied, and also the gene-specific methylation by bisulfite sequencing data of these genes. Based on these results, it was suggested that relatively higher expression and lower level of DNA methylation of *SERK*, *BBM*, *LEC2*, and *WUS* are associated with somatic embryogenesis and plant regeneration [23]. Recently it was reported in cotton that the inhibition of methylation by the use of zebularine activated the transcription of hormone-related genes (*IAA14*, *CKX6*, *LBD1/3*, *LOX1*, *CRF4.1*) and may promote SE [33].

In *Theobroma cacao*, it was reported that the methylation profiles of explants with a lower level of differentiation that generated embryogenic callus (staminoids) were different from the more differentiated tissue profiles (leaves of the explant tree and regenerated plants). It was speculated that these differences could be in the states of differentiation [61]. This information suggests that less differentiated tissues have lower levels of methylation, and are more likely to generate somatic embryos, than those with a higher level of differentiation and higher DNA methylation. A similar hypothesis was formulated for *Daucus carota*, in which it was found that non-embryogenic vacuolar cells contained the highest levels in GDM (25.7%), while meristematic cells (embryogenic tissue) had lower GDM (21.9%) [62]. This work reported that after differentiation and during aging and cell growth, the leaves become more methylated, going from 18.5% in the seedling to 24.0% in the adult plant. Furthermore, it seems that the relationship between hypomethylation and embryogenic potential happens not only in plants but also in trees. Such is the case of three embryogenic cell lines of *Pinus nigra* Arn. ssp. Austriaca with different embryogenic potentials (high, medium or low) [63]. It was found that the line considered to be the most embryogenic (with the capacity to develop the whole embryogenic program and produce plants) showed the lowest levels of DNA methylation, while the least embryogenic line had the highest level of methylation. In another woody species, *Quercus alba*, the methylation levels in embryogenic and non-embryogenic tissues were analyzed [64] and the results were similar to those found in *P. nigra* [63]. The percentage of DNA methylation was significantly lower in embryogenic cells than in non-embryogenic cells, which suggested that DNA methylation decreased during induction of SE. Differences in levels of GDM that depended on the type of tissue (embryogenic or non-embryogenic) have also been reported [64,65]. The analysis showed a lower GDM in somatic embryos, unlike non-embryogenic cell nuclei. Different GDM levels depending on the embryogenic potential suggested that they could be used as markers of early embryogenesis [64]. Furthermore, in *Castanea sativa* [66], it was reported that only the fertilized ovules suffer a decrease in DNA methylation, while the ovules that are not fertilized do not experience this reduction in DNA methylation. Only the ovules that hypomethylated after fertilization are capable of generating zygotic embryos, which suggests that hypomethylation is a pre-requirement to activate the development of zygotic embryos.

Taken together, these reports prompt the hypothesis that if we can find a strategy to manipulate GDM levels, we could improve embryogenic induction in species that have a high level of GDM and low embryogenic potential. Global DNA methylation could be used not only as a marker for the embryogenic capacity of the explant but also to monitor the gradual changes of embryo development. In carrot, globular somatic embryos generate when a reduction in GDM occurs, and this reduction starts to increase during the development and maturation of the somatic embryos [65]. Also in carrot, but using a different system, it was reported that the methylation levels decrease during the change from the somatic to the embryogenic program and in the early stages of the embryos [67]. In later stages of the process, DNA methylation gradually increases. Furthermore, in *C. sativa*, the increase in GDM was also reported during the development of the embryos [66]. Another study found that low levels of GDM were related to the emergence of the proembryogenic mass in *C. canephora* during the SE process, which was also strongly related to the expression of genes involved in cell differentiation [60].

**Figure 3:**
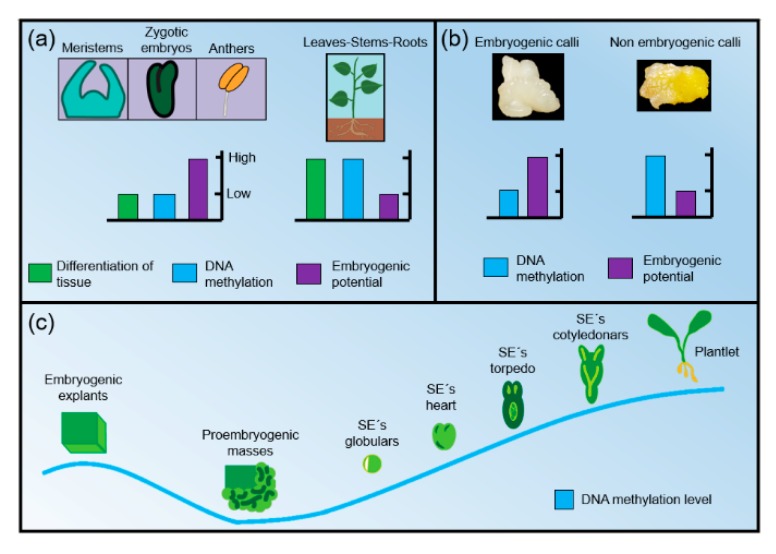
(**A**) Relations between levels of cell differentiation, DNA methylation and embryogenic potential between different kinds of plant tissues used as explants; (**B**) differences in DNA methylation and embryogenic potential between embryogenic and non-embryogenic callus; (**C**) dynamics of DNA methylation levels throughout the SE process.

Based on these findings about DNA methylation and SE response, it is possible that more species follow the same pattern of the dynamics of DNA methylation in different tissues during the process of SE in plants (Figure 3). In summary, it is known that the level of methylation in explants with higher embryogenic potential (usually undifferentiated tissues such as meristems, zygotic or anther embryos) is lower than in explants with less or no embryogenic potential (differentiated plant organs such as stems, roots or leaves). In other words, the embryogenic potential of an explant is inversely proportional to the DNA methylation and the level of tissue differentiation (Figure 3A). Furthermore, the evidence shows that the level of methylation in embryogenic callus is lower than in non-embryogenic callus (Figure 3B) and, when the explants are established in the culture medium (before the induction process), GDM is relatively low. During the induction process, the lowest GDM is observed around the time of the appearance of the first proembryogenic masses/structures. In proembryos and early somatic embryos (preglobular, globulars) there is a gradual increase of GDM. When the embryos are in the late stages (heart and cotyledonar), the GDM is higher and when the embryos germinate and develop into seedlings, the highest level of GDM is achieved (Figure 3C).

### 2.2. The Role of Auxins in DNA Methylation

Several studies have described the relationship between DNA methylation and embryogenic responses, which in some cases depend on the in vitro culture conditions, but mostly are related to the kind and concentration of PGR added to the culture medium [20,65,68,69]. Auxins, especially 2,4-D, are an essential PGR used at the beginning of the SE process. They are known to induce many molecular and metabolic changes that promote the reactivation of cell division and proliferation. 2,4-D is essential in most SE protocols and cannot be removed early in the process; it has been used alone or combined with other PGRs in more than 65% of the protocols for inducing SE [14]. The effect of type and concentration of auxins on genome-wide methylation levels has been studied in several embryogenic cultures. In *D. carota*, it was found that an increase in 2,4-D concentration promotes higher GDM levels [65]. In this work it was reported that SE does not occur when 2,4-D is in the culture medium; however, when it is removed from the medium, the development of somatic embryos is stimulated [65]. A similar process happens in *C. canephora,* but with a different auxin; in this case the plantlets used as a source of explants require a pretreatment period with naphthaleneacetic acid (NAA) and kinetin for two weeks, after which the auxin is removed from the media in order to induce SE [70].

**Figure 4:**
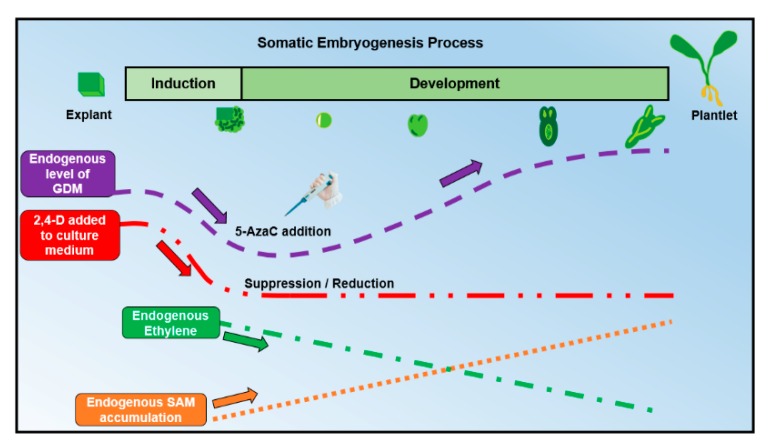
Global DNA methylation, SAM and ethylene dynamics when 2,4-D and 5-AzaC are added to the culture media during the SE process. SAM: S-adenosylmethionine. 2,4-D: 2,4-Dichlophenoxyacetic acid. GDM: global DNA methylation. The purple arrow represents the dynamics of GDM. The red arrow represents the amount of 2,4-D added into the culture medium. The green arrow represents the decrease in endogenous ethylene reported in different studies. The orange arrow represents the increase in the accumulation of endogenous SAM reported in different studies.

It has been suggested that 2,4-D can modify GDM in SE by the accumulation or depletion of S-adenosylmethionine (SAM) and S-adenosylcysteine (SAH) [69]. The removal of 2,4-D produces a reduction in ethylene production (Figure 4), which increases the accumulation of SAM and the SAM/SAH ratio, which in turn causes an increase in DNA methylation [69]. It was observed that an increase in the SAM level is needed to facilitate an increase in the SAM-consuming processes that are necessary for the development of somatic embryos (e.g., SAM-dependent methylations and polyamine biosynthesis), so the availability of SAM may be necessary for the acquisition of embryogenic potential and, later, for the control of embryonic development [71]. Recently, other protocols of SE have reported similar requirements for 2,4-D; e.g., in the date palm [72,73], *Kalopanax septemlobus* [74] and *Coriandrum sativum* [75].

It is likely that the effects observed in the increase or decrease of the embryogenic potential are caused by the up-regulation of genes that encode the transcription factors (TFs) that have a regulatory function in auxin biosynthesis, as reported in the explants of *Arabidopsis thaliana* treated with trichostatin A, an inhibitor of histone deacetylases [76]. Inhibition of methylation caused by 5-AzaC might also alter the expression of TFs involved in auxin or any other PGR biosynthesis, degradation or signaling pathway.

## 3. The Use of 5-Azacytidine During Somatic Embryogenesis

5-AzaC has been used in many protocols of SE for different species of plants (Table 1), among which there have been cases where the embryogenic response has been unquestionably positive, such as in *Pinus pinaster*, *Brassica napus*, *Hordeum vulgare* and *Theobroma cacao.* In *P. pinaster* (Ait.), embryonal masses were exposed to 5-AzaC in different concentrations and durations. When embryonal masses were exposed for 9 days to 5-AzaC, their growth was inversely proportional to the increase in drug concentration. The highest amounts of somatic embryos were obtained at levels of 10 and 15 μM of 5-AzaC [77]. In *Brassica napus* and *Hordeum vulgare* [78], induction of embryos increased with four days of treatment with 5-AzaC (2.5 μM). Similar effects were found in both species, indicating that DNA demethylation promotes the reprogramming of gene expression, acquisition of totipotency and the initiation of embryogenesis in microspores. Embryo differentiation probably requires higher levels of GDM or de novo DNA methylation to acquire a specific pattern of gene expression. In *Theobroma cacao* (cocoa), the embryogenic potential and GDM were analyzed during the long-term secondary SE use as explants from young somatic embryos (12 months of age), aged (36 months of age) and extended SE (39 months), and higher methylation levels were detected in aged somatic embryos [79]. High levels of GDM in long-term SE in cocoa induced a decrease in embryogenic potential, but this decrease was reversed by the addition of 5-AzaC.

Although 5-AzaC has been proven to have positive effects on SE, both the amount and the stage when it is applied need to be taken into consideration to avoid damaging results on the embryogenic process. In *C. canephora*, it was found that when 5-AzaC was added to the culture media in the first seven days of the SE process, the embryogenic response was inhibited [60]. However, when 5-AzaC was added after 21 days of induction, positive effects were observed. The results obtained in this work suggest that the impact of 5-AzaC (mainly at 20 μM) added at day 21 dpi not only synchronized the embryogenic process but also reduced the maturation of the embryo. In hybrid larch (*Laris* x *euroleis*), the addition of 5-AzaC (100 μM) after one week, in multiplication medium, decreased the levels of GDM (from 45.8% to 41.3%) and significantly reduced the relative growth rate of embryonal masses (from 6.3 to 1.8). The value of relative growth rate was obtained by the following formula: ((*FW_t_*_+1_ − *FW_t_*)/*FW_t_*), where *FW* is fresh weight and *t* is time [98]. In another conifer, *Picea omorika*, 5-AzaC (12.3 μM) was added one week before the transfer of the embryogenic explant from the medium of maturation to the proliferation medium [102]. In this case, the numerical values of differential methylation of cytosines (DMC) were obtained by the program ‘RAPD distance 1.04′ and using the algorithm for estimating DNA sequence divergence based on a comparison of restriction endonuclease digests. The DMC value of medium with 5-AzaC (0.267) decreased to 19% compared to the same medium without 5-AzaC (0.323). However, at the end of the experiment, the total number of embryos developed was not significantly different between the control and the treatment with 5-AzaC (181 and 189, respectively).

Recently, a completely inhibitory effect on SE was reported in the model plant *A. thaliana* [34]. In this study, it was reported that in the treatments with 5-AzaC (10 μM) explants produced massively non-embryogenic callus, while in the control (without 5-AzaC) the formation of somatic embryos was fast and efficient. The addition of 5-AzaC reduced the efficiency and productivity of SE and, as a result, only 5% of the explants could undergo SE induction. Since no signs of tissue lethality were observed in the treated cultures, it was hypothesized that the inhibition of SE was not a result of the toxic effect of 5-AzaC on cellular metabolism, but was a consequence of the impact associated with hypomethylation of DNA at the beginning of the process.

### 5-AzaC and 2,4-D Can Work Together During SE

There are important but contradictory studies that describe the effects of the application of 5-AzaC along with 2,4-D in SE protocols. For instance, the addition of 20.5 μM of 5-AzaC + 4.52 mM of 2,4-D in the first 24 h of the SE protocol in *D. carota* generated the same rate of somatic embryo formation as in the control (with the same concentration of 2,4-D but without 5-AzaC) at the end of the process (day 14) [90]. In the same study, it was reported that the formation of somatic embryos at day 14 was severely inhibited when 5-AzaC was applied (without 2,4-D) for three days after the 24-hour treatment. In *Cucurbita pepo* a similar result was reported: the addition of 12.3 μM of 5-AzaC to the basal culture medium (MSC) + 2,4-D caused a statistically significant decrease in DNA methylation in the DEC line (producer of somatic embryos in early stages) and a non-significant reduction in the PEDC line (producer of pro-embryogenic cells). The HEC line (producer of an equal proportion of embryos in all different stages) showed a slight increase in the level of DNA methylation after the addition of 5-AzaC to MSC. The addition of 5-AzaC to MSC with or without 2,4-D did not significantly alter the proportion of embryos in different stages compared to that found in the same medium without 5-AzaC [87]. On the other hand, in *Acca sellowiana*, the effect of 5-AzaC and 2,4-D on GDM during SE showed that a pulse of 2,4-D (200 μM) + AzaC (50 μM) generates an increase in GDM and improves the induction of SE [81].

We broadly discussed above that auxins are hypermethylating substances, while 5-AzaC has a hypomethylating effect. However, because both substances have been used in different concentrations, in different proportions (2,4-D/5-AzaC) and at different times (without mentioning the difference between SE protocols and species studied), it is difficult to determine the optimal balance to obtain the greatest embryogenic potential in SE protocols. One aspect that we can highlight in the three studies cited above (*D. carota*, *C. pepo*, and *A. sellowiana*) is the moment where 5-AzaC was added. In *C. pepo* 5-AzaC was added to the medium where the embryogenic calluses already induced were established before they formed somatic embryos. In *Acca sellowiana*, 5-AzaC was added as a pretreatment and had positive effects on embryo generation. In the first two studies (*D. carota* and *C. pepo*) where 5-AzaC was added late to the process, the results were visibly adverse. On the other hand, in *A. sellowiana,* 5-AzaC was added at the beginning of the process and had positive effects. This suggests that the time at which 5-AzaC is added is determining in the outcome of the process: the earlier it is added, the less negative the effects on SE will be.

Therefore, based on the SE systems in which the use of 5-AzaC is reported, one needs to do some preliminary experiments to ensure reproducible results with the literature. These are the most important:

- Test the effects of different concentrations of 5-AzaC to know the minimum levels to observe the differential impact and maximum concentrations so that they are not toxic to the explants.
- Select the timing of the process for adding the inhibitor, as the effect could make the embryogenic process more efficient or inhibit it, depending on whether 5-AzaC is applied before/during the induction or development of the somatic embryos.
- If the culture medium includes reagents that sequester substances, such as activated carbon [111], higher concentrations of the inhibitor should be applied than in culture media without this type of reagent. Another option is to use a pre-treatment with the inhibitor for a specific time and then transfer the explant to the conventional culture medium if it contains activated charcoal.
- Take into consideration the pH and temperature of the culture medium at the time the inhibitor is applied. It has been reported that 5-AzaC is moderately stable in acidic solutions while rapidly decomposing in alkaline media and that degradation is accelerated dramatically with increasing temperature [112].

Based on all of the information previously discussed, we propose an optimal time to add 5-AzaC (Figure 4). Because there is a direct relationship between the development of SE and the levels of GDM, it is essential to know the three common phases in which GDM has the most significant influence. Before the SE induction process starts, explants exhibit elevated levels of GDM (probably due to the high concentrations of 2,4-D used in the culture media to induce SE, which also affects the endogenous production of ethylene and the SAM/SAH ratio [69]). Then, during the induction process (specifically in the phase of acquisition of embryogenic competence), GDM reaches its lowest levels in pro-embryogenic cells. Finally, GDM gradually increases from the phase of globular somatic embryos to the seedling phase. Thus, we can hypothesize that 5-AzaC addition could help to reduce GDM in the induction phase, promoting the establishment of an optimal level of methylation (mostly low). This would trigger in the explant cells the acquisition of embryogenic competence. As SE progressed, 5-AzaC would be assimilated and later degraded, so that the normal increase of GDM required for SE would follow its ordinary path in advanced developmental stages.

## 4. Conclusions

Low levels of DNA methylation are related to high embryogenic potential in explants during the induction of the SE. However, it is still necessary to generate more information on other SE protocols (by direct and indirect pathways) and make a more methodical comparison between mono- and dicotyledonous plants to corroborate this pattern.

When such SE studies are available, it would be beneficial to study the biochemical, molecular and epigenetic changes that accompany the acquisition or loss of morphogenic competence. In these protocols (as in the vast majority of protocols that have been reported over the years in various species), cultures need a high concentration of 2,4-D in the induction process. This auxin needs to be reduced (or totally removed) to promote the development of somatic embryos. Moreover, the 5-AzaC addition helps to reduce GDM in the induction phase, improving the establishment of an optimal level of methylation (low) to be carried out in the explant cells’ acquisition of embryogenic competence. With the use of 5-AzaC in SE protocols, it is possible to demonstrate that DNA methylation plays a significant role in the acquisition of embryogenic competence of plant cells. In the near future, there will be more accurate information about cellular processes that are directly affected by DNA methylation during SE.

The DNA methylation level in several systems that have been used 5-AzaC to promote SE has been determined with different methods such as HPLC, ELISA, MSAP and others (Table 1). However, the selected methodology depends on the kind of DNA methylation to be analyzed and the information that is needed to answer a specific biological question. Each method can give information about DNA methylation such as global DNA methylation, regional DNA methylation, genome analysis, methylation analysis DNA sequencing, detection of particular methylation patterns and individual CpG analysis (Table 1) [21].

## Figures and Tables

**Table 1:** Techniques used to evaluate DNA methylation and the effect of methylation inhibitors on the somatic embryogenesis (SE) of different species of plants.

Species	Family	Detection of DNA Methylation (Method)	DNA Methylation Inhibitor Used	Effects of Inhibitor	References
*Acca sellowiana*	Myrtaceae	CRED-RA	NA	NA	[80]
		HPLC	5-AzaC	5-AzaC (50 μM) induced an increase in GDM and improved the induction of SE. However, in the conversion phase, somatic embryos had a deregulatory effect during the formation of autotrophic plants, resulting in significantly lower conversion rates	[81]
*Arabidopsis thaliana*	Brassicaceae	ELISA	5-AzaC	Explants treated with 5-AzaC (10 μM) showed a drastic inhibition of SE and the explants produced massive non-embryogenic callus, whereas in non-treated-explants they formed somatic embryos quickly and efficiently	[34]
*Araucaria angustifolia*	Araucariaceae	HPLC	NA	NA	[82]
*Bactris gasipaes*	Arecaceae	HPLC	NA	NA	[83]
*Brachypodium distachyon*	Poaceae	TUNEL	5-AzaC	At a concentration of 50 µM of 5-AzaC, induction of embryogenic masses (EM) was totally inhibited, while in 5 µM of 5-AzaC 10% of explants (zygotic embryos) developed callus with EM.	[84]
*Brassica napus*	Brassicaceae	ELISA	5-AzaC	Induction of embryos increased when explants were treated four days in 5-AzaC (2.5 μM). In longer treatments with 5-AzaC the formation of somatic embryos decreased	[78]
*Castanea sativa*	Fagaceae	HPCE	NA	NA	[66]
*Citrus paradise*	Rutaceae	MSAP	NA	NA	[85]
*Coffea canephora*	Rubiaceae	HPLC	5-AzaC	Embryogenic process was strongly inhibited when 5-AzaC was added earlier. However, this negative effect was not observed when added to the 35 days post induction (dpi). The effect of 5-AzaC (20 μM) added at day 21 dpi not only synchronized the embryogenic process but also reduced the maturation of somatic embryos	[60]
		MSAP	NA	NA	[86]
*Cucurbita pepo*	Cucurbitaceae	MSAP	5-AzaC	Addition of 5-AzaC (12.3 μM) to the basal medium (MSC) with or without 2,4-D did not significantly alter the proportion of embryos in different stages compared to that found in the same medium without 5-AzaC. In the MSC medium with 2,4-D and 5-AzaC, most embryos remained in the early stages of development; however, some developed to more mature stages	[87]
		CRED-RA/MSAP	5-AzaC	5-AzaC had no effects (global DNA methylation or capacities for the development and regeneration) on embryogenic cultures	[88]
*Daucus carota*	Apiaceae	HPLC	5-AzaC/ECP	When ECP is added, SE is immediately blocked. Isolated mutant line that is resistant to the hypomethylating activity of ECP and 5-AzaC shows a higher level of endogenous indole acetic acid (IAA) and a different metabolism of IAA, suggesting the endogenous synthesis of IAA in the habituated tissue could be the reason for its low sensitivity to methylation inhibitors	[65]
		Immunodetection	5-AzaC	5-AzaC suppresses embryogenesis but does not prevent the proliferation of dedifferentiated cells from cells in suspension.	[89]
			5-AzaC	When 5-AzaC (0.41 μM) was added to the medium, somatic embryos were formed to the same extent as in the control without 5-AzaC. When 5-AzaC (20.5 μM) was supplemented for 3 days after the 24-hour treatment with 2,4-D, the formation of somatic embryos was severely inhibited	[90]
		HPLC	NA	NA	[62]
*Elaeis guineensis*	Arecaceae	HPLC/MSAP	NA	NA	[91]
		HPLC	NA	NA	[92]
*Eleuterococcus senticosus*	Araliaceae	HPLC/MSAP	NA	NA	[93]
*Freesia hybrida*	Iridaceae	MSAP	NA	NA	[94]
*Gentiana pannonica*	Gentianaceae	HPLC	NA	NA	[95]
*Hordeum vulgare*	Poaceae	MS-AFLP	NA	NA	[96]
		ELISA	NA	NA	[67]
		ELISA	5-AzaC	Induction of embryos increased with four days of treatment with 5-AzaC (2.5 μM), the response was associated with a decrease in DNA methylation. In contrast, longer 5-AzaC treatments decreased embryo generation	[78]
		HPLC/MS-AFLP	NA	NA	[97]
*Laris x eurolepis*	Pinaceae	HPLC	5-AzaC/Hydroxy-urea	5-AzaC (100 μM) altered the overall DNA methylation status of embryogenic cultures and significantly reduced their relative growth rate and embryogenic potential	[98]
*Medicago truncatula*	Fabaceae	MSAP	5-AzaC	5-AzaC (100 μM) stopped the generation of somatic embryos in the embryogenic line and the proliferation of callus in the non-embryogenic line. Analysis with restriction enzymes sensitive to total DNA methylation extracted from the untreated 5-AzaC-treated callus showed a decrease in DNA methylation levels	[99]
*Ocotea catharinensis*	Lauraceae	MSAP	NA	NA	[100]
*Pennisetum purpureum*	Poaceae	HPLC/MSAP	NA	NA	[101]
*Picea omorika*	Pinaceae	MS-RAPD	5-AzaC	DNA methylation decreased by 19% compared to the same medium without 5-AzaC (12.3 μM). However, the total number of embryos developed in the subsequent transfer to the maturation medium was not significantly different (182 and 190 somatic embryos, respectively)	[102]
*Pinus nigra*	Pinaceae	CRED-RA	NA	NA	[63]
*Pinus pinaster*	Pinaceae	HPLC	5-AzaC	Embryonal masses grew when they were exposed 9 days to 5-AzaC. Growth was inversely proportional to the increase in drug concentration. The highest amounts of somatic embryos were obtained at the 10 and 15 μm concentrations of 5-AzaC, the treatments with the highest levels of methylation (19.5% and 21.3%, respectively)	[77]
*Quercus alba*	Fagaceae	ELISA	NA	NA	[64]
*Quercus suber*	Fagaceae	HPCE/Immunolocalization	NA	NA	[103]
*Rosa hybrida*	Rosaceae	MS-AFLP	NA	NA	[104]
*Solanum tuberosum*	Solanaceae	MS-AFLP	NA	NA	[105]
*Theobroma cacao*	Malvaceae	MSAP	NA	NA	[61]
		MSAP	NA	NA	[106]
		HPLC	5-AzaC	GDM increased as SE proceeded and during the extended SE the aged somatic embryos could recover embryogenic potential when treated with 5-AzaC (20 μM). The results of this study suggested that long-term SE in cocoa induced a decrease in embryogenic potential, but that it could be reversed by 5-AzaC supplementation	[79]
*Triticosecale*	Poaceae	HPLC	NA	NA	[107]
*Vitis vinifera*	Vitaceae	MSAP	NA	NA	[108]
*Zea mays*	Poaceae	MSAP	NA	NA	[109]
		meDIP	NA	NA	[110]

*5-AzaC* 5-Azacytidine, *CRED-RA* Coupling of Restriction Enzyme and Random Amplification, *ELISA* Enzymatic-Linked Immunosorbent Assay, *HPCE* Hight-Performance Capillary Electrophoresis, *HPLC* High-Performance Liquid Chromatography, *meDIP* Methylated DNA Immunoprecipitation, *MS-AFLP* Methylation Sensitive—Amplification Fragment Length Polymorphism, *MS-RAPD* Methylation Sensitive—Random Amplification of Polymorphic DNA, *MSAP* Methyl-Sensitive Amplification Polymorphism, *TUNEL* Terminal deoxynucleotidyl transferase dUTP Nick End Labeling, *ECP* 2-amino-5-ethoxycarbonyl-pyrimidine-4 (3H), *NA* Not Applied.
